# Recent Advances in Sample Preparation and Chromatographic/Mass Spectrometric Techniques for Detecting Polycyclic Aromatic Hydrocarbons in Edible Oils: 2010 to Present

**DOI:** 10.3390/foods13111714

**Published:** 2024-05-30

**Authors:** Jiayi Gao, Xingyue Li, Yuanyuan Zheng, Qian Qin, Di Chen

**Affiliations:** 1 Key Laboratory of Targeting Therapy and Diagnosis for Critical Diseases of Henan Province, School of Pharmaceutical Sciences, Zhengzhou University, Zhengzhou 450001, China; 2 College of Medical Laboratory, Dalian Medical University, Dalian 116044, China; 3 Zhengzhou Research Base, National Key Laboratory of Cotton Bio-Breeding and Integrated Utilization, Zhengzhou University, Zhengzhou 450001, China

**Keywords:** polycyclic aromatic hydrocarbons, edible oils, sample preparation, mass spectrometry, gas chromatography, liquid chromatography

## Abstract

Polycyclic aromatic hydrocarbons are considered to be potentially genotoxic and carcinogenic to humans. For non-smoking populations, food is the main source of polycyclic aromatic hydrocarbons exposure. Due to their lipophilic nature, oils and fats rank among the food items with the highest polycyclic aromatic hydrocarbon content. Consequently, the detection of polycyclic aromatic hydrocarbons in edible oils is critical for the promotion of human health. This paper reviews sample pretreatment methods, such as liquid-phase-based extraction methods, adsorbent-based extraction methods, and the QuEChERS (quick, easy, cheap, effective, rugged, and safe) method, combined with detection techniques like mass spectrometry and chromatography-based techniques for accurate quantification of polycyclic aromatic hydrocarbons in edible oils since 2010. An overview on the advances of the methods discussed herein, along with a commentary addition of current challenges and prospects, will guide researchers to focus on developing more effective detection methods and control measures to reduce the potential risks and hazards posed by polycyclic aromatic hydrocarbons.

## 1. Introduction

Polycyclic aromatic hydrocarbons (PAHs) constitute a significant category of carcinogenic chemicals and are among the most concerning compounds [1]. Composed of over 200 different compounds, PAHs are hydrophobic organic molecules consisting of two or more densely packed aromatic rings [2]. For non-smoking populations, food is the primary source of PAH exposure [3], with fats and oils being particularly rich in PAHs, due to their lipophilic nature [4,5]. It has been reported that 15–50% of PAH intake from dietary sources is attributed to contaminated oils and fats [6]. Annually, over 170 million tons of edible oil are consumed globally [7]. The contamination of edible oils with PAHs can occur through several pathways, including the contamination of raw materials, seed drying with combustion gases prior to oil extraction, solvent extraction, soil combustion, packaging materials, mineral oil residues, and migration from contaminated water or soil [4]. Additionally, the oxidation of edible oils during extended storage can also lead to PAH formation [8].

Once ingested, PAHs are metabolically activated by cytochrome P450 enzymes, forming electrophilic substances that bind covalently to DNA molecules, causing mutations and potential genotoxic effects [9]. Generally, the toxicity of PAHs increases with the number of aromatic rings in their molecular structure [10]. In 2012, the International Agency for Research on Cancer classified benzo(a)pyrene (BaP) molecule, a specific type of PAH, as a human carcinogen [11]. Due to their hazardous effects, monitoring and controlling PAH levels in edible oils is critical. In 1970, the U.S. Environmental Protection Agency (EPA) identified 16 PAHs as priority pollutants requiring routine monitoring, listed in Table 1 [12]. In 2002, the European Commission’s Scientific Committee on Food identified an additional 15 PAHs as carcinogens or mutagens, with only 8 aligning with EPA regulations [13]. The addition of benzo(c)fluorene in 2005 led to the establishment of the European Union (EU) 15+1 priority PAHs (BaA, Chr, BbF, BaP, BkF, DBahA, BghiP, IP, Cpp, BcFl, 5-MChr, BjFA, DBaiP, DBaeP, DBalP, DBahP, and BeP), and BaP was recognized as a marker for carcinogenic PAHs in food [14]. However, the European Food Safety Authority concluded in 2008 that a group of four (benzo(a)anthracene, benzo(b)fluoranthene, chrysene, and BaP) or eight PAHs (including benzo(k)fluoranthene, dibenzo(a,h)anthracene, benzo(g,h,i)perylene, and indeno(1,2,3-cd)pyrene) provide a more accurate indication of PAH levels than BaP alone [15]. Subsequently, the EU set the maximum allowable concentrations of PAHs in vegetable oils at 10 μg/kg and BaP at 2 μg/kg, while the limit for coconut oil was set at 20 μg/kg [16]. In 2012, China established a maximum level of BaP in oils at 10 μg/kg [17].

Recent advancements have not only significantly improved the techniques for sample preparation, but also enhanced the efficiency and accuracy of chromatographic and mass spectrometric methods used to detect PAHs in edible oils. From 2010 to the present, there has been a noteworthy shift towards developing more sensitive, faster, and more environmentally friendly methods. This review aims to not only summarize these technological advancements, but also to highlight their impact on the regulatory landscape and public health. By providing a detailed analysis of these evolving techniques, this paper seeks to inspire further research and development in the field. The ultimate goal is to establish more robust and adaptable analytical methods that can keep pace with the increasing complexity of food contamination and the evolving regulatory requirements aimed at protecting consumers from the risks associated with PAH exposure.

The literature retrieval was primarily conducted through the Web of Science and PubMed databases, with the search timeframe set from 2010 to 2024, focusing on the topic of PAHs. The search was refined by including the keyword “oil”, followed by additional terms such as “sample preparation”, “determination”, and “analysis” to filter the selected publications, specifically excluding review articles. The relevant studies concerning the detection of PAHs in edible oils using chromatographic and mass spectrometry techniques were imported into EndNote X9. Supplementary searches were performed using Scopus and Google Scholar databases to enrich the literature base, establishing a solid foundation for the subsequent analysis.

## 2. Sample Preparation Methods

Given the complexity of edible oil matrices, efficient sample preparation is essential to extract target analytes prior to detection [18]. Sample preparation typically involves steps such as sample collection, saponification, extraction, purification, and concentration of edible oils. The primary pretreatment methods for detecting PAHs in edible oils include saponification, gel permeation chromatography (GPC), liquid-phase-based and adsorbent-based extraction methods, and the QuEChERS method.

### 2.1. Saponification

For accurate PAH detection in edible oils, it is crucial to remove lipophilic matrix compounds such as triglycerides [19]. These substances can contaminate the detector, column, or inlet of the chromatographic system, leading to inaccurate measurements and potential equipment damage [20]. Saponification is an effective method for eliminating these compounds from the sample matrix. Typically, edible oil samples are treated with a methanol KOH (e.g., 4 mol/L) solution, followed by two to three extractions with hexane or toluene [21]. Of course, KOH solution can be replaced with other alkalis, such as NaOH solution. Through experiments, Wang et al. [21] found that, when using 4 mol/L, 6 mol/L, and 8 mol/L KOH for saponification reactions as pre-treatments for edible oils, the recovery rates of BaP (benzo[a]pyrene) could reach 94%, 93%, and 91%, respectively. However, the alkali used in the saponification process is generally dissolved in an organic solvent like methanol [22]. To facilitate the reaction in an alkaline aqueous solution and minimize the use of organic solvents, Li et al. [23] utilized the powerful tool of phase transfer catalysts (PTCs). PTCs can accelerate chemical reactions between mutually insoluble species (hydrophobic and hydrophilic substances) at the oil–water interface. They employed phase transfer catalysts, such as tetrabutylammonium bromide, which effectively transferred substances from the oil phase to the aqueous phase and accelerated the reaction of the insoluble substances at the oil–water interface. This approach reduced the use of organic solvents and enhanced the overall efficiency of the saponification process.

### 2.2. Gel Permeation Chromatography

GPC leverages the size-exclusion properties of porous gels to separate molecules of different sizes. In this process, a sample is carried by the mobile phase through a porous gel column, where molecules traverse the gaps between the gel particles. Larger molecules have shorter migration paths and elute from the column first, while smaller molecules diffuse deeper into the gel particles and elute later, thus achieving separation. Specifically, Liu et al. [24] sonicated a 2-g sample of vegetable oil with 10 mL of a vinyl acetate:cyclohexane (1:1, *v*/*v*) solution. For cleanup, they used a Bio-Beads S-X3 gel as the stationary phase and the same vinyl acetate, with a cyclohexane mixture as the mobile phase at a flow rate of 5 mL/min. The eluate, containing the target PAHs, was collected from 19 to 50 min for subsequent evaporation and high-performance liquid chromatography with a fluorescence detector (HPLC-FLD) analysis of 15 PAHs (BaA, Chr, BbF, Bap, BkF, DahA, BghiP, Icdp, Pyr, Flt, Ant, Phe, Flo, Ace, and Nap) in Chinese vegetable and frying oils. However, the GPC extracts may still contain matrix compounds that could interfere with the analytes of interest. Traditionally, GPC fractionation is combined with solid-phase extraction (SPE) or magnetic solid-phase extraction (MSPE) to improve purity [25]. Conventional GPC techniques suffer from low sample loading capacity, high solvent consumption, and they are labor-intensive and time-consuming [19]. To address these limitations, Wang et al. [26] developed a custom, narrow and short GPC column that significantly reduced both solvent use (approximately 60 mL) and GPC resin use. This setup, combined with isotope dilution gas-chromatography–mass-spectrometry (GC-MS), enabled the determination of 16 EPA priority PAHs in edible oils with quantitation limits below 0.5 ng/g. Furthermore, Cotugno et al. [27] reported a state-of-the-art dual-column GPC system that increased the sample loading capacity by a factor of seven compared to a single column. This innovation improved both the limits of detection (LODs of 0.21–0.32 ng/g) and the limits of quantification (LOQs of 0.70–1.06 ng/g), markedly reducing the analysis time and solvent use while enhancing the sensitivity of PAH detection in olive oil.

### 2.3. Liquid-Phase-Based Extraction Methods

Liquid–liquid extraction (LLE) is a conventional sample preparation method that involves transferring target analytes from the sample solution to an extractant. This transfer is based on differing partition coefficients between two immiscible or slightly soluble solvents. Commonly used extraction solvents include n-hexane [28], acetonitrile [29], a mixture of acetonitrile and acetone [30,31], dichloromethane [32], and dimethylacetamide [33]. Table 2 provides a summary of the methods employed to analyze PAHs in edible oils using LLE in combination with appropriate detection techniques.

However, LLE is typically time-consuming, requires substantial amounts of organic solvents, poses environmental risks, and often results in low enrichment factors. Consequently, there is an increasing interest in microextraction techniques that streamline the sample preparation process [37]. Liquid-phase microextraction (LPME) is a miniaturized version of LLE that significantly reduces the amount of organic solvent used [43]. Despite its advantages, traditional organic solvents often pose challenges such as toxicity, volatility, and environmental pollution, thus highlighting the need for greener alternatives. Adib et al. [37] synthesized a novel deep eutectic solvent ([Ch: 4-chlorophenol] + [FeCl_4_]^−^) for use in a surfactant-enhanced air liquid–liquid microextraction procedure, successfully extracting PAHs from edible oils. This method was evaluated using high-performance liquid chromatography with a diode-array detector (HPLC-DAD), achieving a score of 81 on the Analytical Eco-Scale, indicating a highly green analytical method (with scores of over 75 being considered excellent). This method offers environmental benefits, a broad linear range, and low LODs and LOQs, with the values of 0.04–0.13 ng/g and 0.13–0.43 ng/g, respectively.

**Figure 1:**
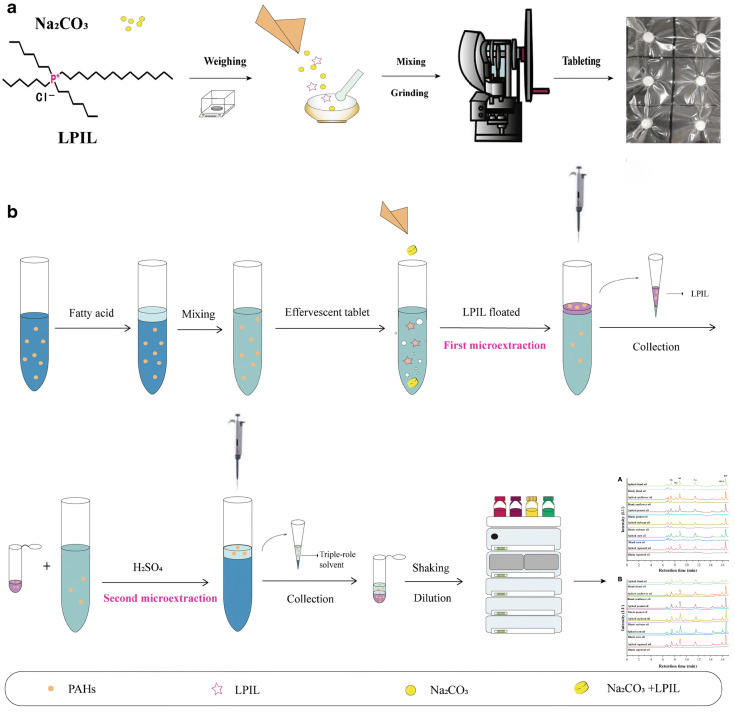
Schematic diagram of the EM-LPSH procedures. (**a**) Preparation of effervescent tablets. (**b**) Operational processes for the EM-LPSH method. (**A**) Unheated edible oil. (**B**) Heated edible oil. Reproduced from [35], with permission from Jing, Q, 2021.

Another widely used technique in LPME is dispersive liquid–liquid microextraction (DLLME), which facilitates the contact area between the extractant and the analyte by forming dispersed droplets in the sample solution with the aid of a dispersant. This process accelerates the achievement of an equilibrium between the sample solution and the extractant [44]. However, the effectiveness of DLLME is often constrained by the microliter volume of the extraction solvent and the tendency of organic droplets to aggregate at the bottom of the centrifuge tube, as the solvents used are denser than water [45]. To address these issues, Jing et al. [35] introduced a novel lighter-than-water phosphine-based ionic liquid as the primary extractant, coupled with a switchable hydrophilic-hydrophobic fatty acid that serves as both a dispersant and an acid source in the effervescent microextraction reaction (Figure 1). This innovative approach eliminates the requirement for additional mechanical agitation to disperse the extractant, simplifying the process and enhancing the overall efficiency of PAH extraction from edible oils.

### 2.4. Adsorbent-Based Extraction Methods

Solid-phase extraction (SPE) remains a highly favored method for sample preparation, due to its straightforward operation, high enrichment capability, and minimal solvent usage. SPE is particularly prevalent in the analysis of PAHs in edible oils. This technique involves passing a complex sample solution through an extraction column filled with adsorbent, where target compounds are retained while impurities are flushed out using selective solvents for effective separation. Recent advancements have seen SPE employed in conjunction with various analytical methods to enhance PAH extraction from edible oils, as detailed in Table 3.

Typically, SPE requires either pre-dilution of oil or LLE [41,73], utilizing adsorbents like C18 [8], Florisil [28], silica [10,33], humic-acid-bonded silica [75], and alumina [77]. In pursuit of a more streamlined approach, Stenerson et al. [61] developed a method involving direct loading of undiluted olive oil onto a dual-layer SPE column incorporating Florisil, zirconium-oxide-coated silica, and C18. This was followed by elution with acetonitrile, concentrating the extract for direct analysis using GC-MS or HPLC-FLD. Remarkably, this method not only reduces procedural steps and solvent usage, but also achieves high sensitivity. The LODs for PAHs ranged from 0.2 to 1 µg/kg, and the LOQs were between 0.65 and 3.4 µg/kg.

In SPE, the nature of the adsorbent plays a crucial role in the adsorption of analytes. However, conventional adsorbents have a limited application to some extent, due to their low selectivity and adsorption properties. Therefore, the development of novel adsorbents has been a hot topic of research in this field. Farrokhzadeh et al. [46], for the first time, used chicken-flaw yellow membrane (CFYM) as a novel biosorbent for the extraction and enrichment of organic pollutants. They successfully detected four PAHs (Phe, F, P, A, and Na) in edible vegetable oils using CFYM in combination with HPLC and ultraviolet absorption detector (UV). This natural adsorbent has the advantages of simple preparation, safety, low cost, and being environmentally friendly and easy to utilize compared to conventional chemical adsorbents. The low LODs of 0.37–38.5 ng/kg, wide linear range, suitable recovery, good reproducibility, and reusability make it a green method for the analysis of PAHs in edible oils.

Solid-phase microextraction (SPME) technology based on SPE uses a polymer coating on quartz glass fiber or adsorbent as an adsorption medium to extract and enrich the organic molecules in the sample [79]. It is a new sample preparation technique that integrates the steps with adoption, extraction, enrichment, and injection into one process, with the advantages of being solvent-free and pollution-free, requiring a low sample dosage, being easy to reuse, and aligning with the concept of green chemistry [69,80]. Currently, although many commercial SPME fibers are available, the coatings are easily peeled off, the fibers are easily broken, and the selectivity and adsorption capacity of the fibers are limited due to their thermal, mechanical, and chemical stability. To overcome these drawbacks, researchers have been searching for novel fibers with high extraction efficiency, low cost, and thermal stability. Hasanli et al. [54] prepared a novel porous nanostructured SPME fiber (SNF@Cu) using a fast and simple galvanic displacement reaction, which was applied in conjunction with GC-MS for the determination of PAHs in sunflower oil. The LODs of the method were 0.1–1.2 ng/mL, and the LOQs were 0.3–3.6 ng/mL. The extraction effect of SNF@Cu on PAHs was compared with that of commercial fibers, and the results showed that its LODs were comparable to that of commercial fibers, but it had higher thermal and chemical stability, and it was easy to prepare, rapid, and inexpensive. In some complex samples, the sample matrix may interfere with the target analytes, which can affect the extraction efficiency. Headspace solid-phase microextraction (HS-SPME) offers a solution to this challenge. By elevating the temperature, HS-SPME facilitates the release of compounds from the sample matrix into the headspace of the vial, where they are then captured by the SPME coating. This method prevents direct contact with the sample solution, thus avoiding coating contamination and effectively overcoming several limitations of traditional solid-phase microextraction techniques in specific applications. Liu et al. [59] used headspace solid-phase microextraction coupled with GC-MS for the detection of 1-methylnaphthalene in vegetable oils.

**Figure 2:**
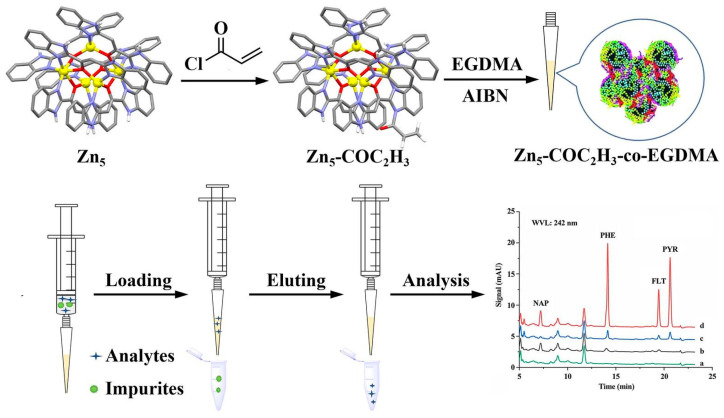
Schematic of the synthesis of Zn_5_ copolymer monolithic column and μ-SPE procedure. Adapted from [60], with permission from X. Wu, 2023.

Micro-solid-phase extraction (μ-SPE) is a new type of SPE method that has the advantages of using a small amount of organic solvent, easy operation, rapidity, and having wide range of application. The physical and chemical properties of the adsorbent directly affect the extraction efficiency and selectivity of μ-SPE [81]. Wu et al. [60] used a synthesized Zn5 copolymer monolithic column for μ-SPE to establish a μ-SPE-HPLC-UV method for the determination of four PAHs (P, F, Phe, and Na) in edible oils (Figure 2). The results showed that the chemical modification of the Zn5 clusters enabled the monolithic column to have abundant adsorption sites for the four PAHs, which extended the service life, and the copolymerization promoted the development of the composite monolithic column for the determination of the four PAHs. The recoveries of the four PAHs ranged from 86.3 to 101.5%, with LODs of 0.050–1.5 μg/L and LOQs of 0.15–5.0 μg/L. Nowadays, the automated μ-SPE has greatly simplified the extraction process, achieving a fast workflow, low organic solvent usage, and low manual labor, thus reducing costs. Eyring et al. [19] used GC with quadrupole orbital MS of a Triplus autosampler for automated clean-up analysis of PAHs in sunflower seed oil.

A significant advantage of dispersive solid-phase extraction (d-SPE) over SPE is the ability to increase the volume of the sample without prolonging the extraction time, overcoming the limitations of SPE that can be encountered with cartridge clogging and clogging of the active sites on the adsorbent. As a result, d-SPE has gained rapid acceptance as a method for analyte enrichment or sample purification [82]. Adsorbents, which usually have a very high surface area, can easily adsorb and extract analytes to a large extent. In this regard, multi-walled carbon nanotubes (MWCNTs) have an affinity for planar aromatic compounds and offer great potential for the detection of PAHs in edible oils [52]. Zacs et al. [53], for the first time, used MWCNTs as d-SPE adsorbents for the extraction of four PAHs (BaA, Chr, BbF, and BaP) in edible oils, thus eliminating the saponification technique, in combination with GC-MS/MS. The LODs and LOQs of the four PAHs ranged from 0.06 to 0.21 μg/kg and from 0.19 to 0.71 μg/kg, respectively, with spiked recoveries in the range of 98–108%. The method provided almost quantitative recoveries of analytes, with minimal presence of co-extractables in the final extract.

**Figure 3:**
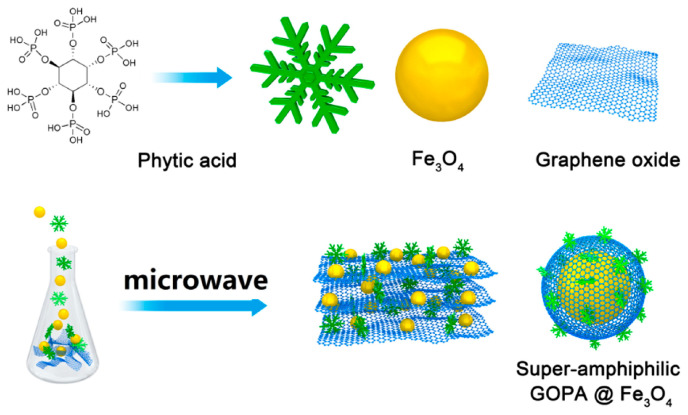
Schematic for the preparation of super-amphiphilic GOPA@ Fe_3_O_4_. Adapted from [62], with permission from W. Ji, 2017.

MSPE uses magnetic or magnetizable materials as adsorbents to adsorb the target analytes onto the surface of the dispersed magnetic adsorbents. By applying an external magnetic field, the adsorbents and analytes can be easily and quickly separated from the solution. The target analytes can then be eluted using suitable solvents [83]. MSPE simplifies the sample preparation process by eliminating the need for filtration and centrifugation. It also avoids common problems associated with traditional SPE packing materials, such as high backpressure of adsorbents, clogging, and non-regeneration. MSPE has garnered considerable attention as a sample preparation method for the high selectivity and high throughput enrichment of PAHs. Commonly used adsorbents include covalent organic frameworks [51], nanofibers [58], graphene oxide (GO) [23], carbon nanotubes [63], and various other magnetic composites. To overcome the problem of the inhomogeneous adsorption of magnetic GO adsorbents in hydrophobic media, Ji et al. [62] prepared super amphiphilic GOPA@Fe_3_O_4_ adsorbent based on the stability of phytic acid for the MSPE procedure of PAHs in vegetable oils (Figure 3). This method was combined with HPLC-DAD for the determination of PAHs in vegetable oils. The method showed a wide linear range (0.2–200 ng/g), high precision (3.44–8.41%), LODs of 0.06–0.15 ng/g, and spiked recoveries of 85.6–102.3%.

### 2.5. QuEChERS

QuEChERS, developed by the United States Department of Agriculture, Anastassuades et al. [84], in 2003, is a recently advanced international rapid sample preparation technique for the detection of agricultural products. Due to its simplicity, low cost, and relatively high efficiency, it has been widely used for the detection of PAHs in edible oils. Table 4 summarizes the method of analyzing PAHs in edible oils through QuEChERS, combined with suitable detection technology. A typical QuEChERS process consists of two steps, as follows: salting-out extraction (anhydrous sodium sulfate, acetonitrile, etc.), followed by the removal of impurities and purification by d-SPE. Sun et al. [20] were the first to use freezing-lipid precipitation in combination with QuEChERS for the extraction of PAHs in edible oils. They performed the LLE method with 2 g of mixed edible oil and acetonitrile-saturated hexane, followed by freezing-lipid precipitation, and finally purification by a d-SPE procedure using Z-Sep+-C18 as the extractant. This method improved the accuracy by effectively removing the lipids and prevented the lipids from contaminating the inlet and column of the GC-MS. In QuEChERS optimization, the choice of adsorbent is critical to reduce matrix interference in chromatographic analysis. Notably, enhanced matrix-removed lipids (EMR-lipids) exhibit superior lipid adsorption capacity and have been designed for the analysis of PAHs in edible oils. Sun et al. [85] used EMR-lipids as a dispersed solid-phase adsorbent and a mixture of acetonitrile and acetone (3:2, *v*/*v*) as the extraction solvent to establish a QuEChERS-GC-MS method for the determination of 16 PAHs (Na, Acy, Ace, F, Phe, A, Fl, P, BaA, Chr, BbF, BkF, BaP, IP, DBahA, and BghiP). The LODs of the method were 0.06–0.13 μg/kg and the recoveries of heavy PAHs fluorene, benzo(a)anthracene, and benzo(b)fluoranthene were improved by 12.51–34.33%.

## 3. Detection Techniques

### 3.1. Mass Spectrometry

MS involves the ionization of the components of the sample in an ionizer, generating charged ions with different mass-to-charge ratios. These ions are accelerated by an electric field, forming an ion beam that enters a mass analyzer, such as a quadrupole rod, a time-of-flight tube, or a magnetic sector mass spectrometer [88]. Under the action of the electric and magnetic fields, the ions with different mass-to-charge ratios undergo velocity dispersion and eventually focus on the detector to form a mass spectrum. Due to its unrivaled sensitivity, specificity, and ability to provide structural information, MS has become an indispensable analytical technique for the determination of PAHs in edible oils. 

Recently, matrix-assisted laser desorption ionization time-of-flight mass spectrometry (MALDI-TOF MS) has gained attention, due to its high throughput, high sensitivity, short experimental period, and low sample consumption. However, applying MALDI-TOF MS to small molecules (less than 600 Da) can be challenging. Wang et al. [34] successfully determined BaP in sesame oil, flaxseed oil, camellia seed oil, and olive oil using metal–organic framework MIL-101(Fe) as a matrix by MALDI-TOF MS technique. The method was developed with an analysis time of only 1 min, a LOD as low as 0.1 μg/L, and a recovery range of 80.0–114.8%. Although MALDI-TOF MS exhibits excellent selectivity and sensitivity, it has the drawback of a cumbersome sample preparation process. Paper spray ionization mass spectrometry (PSI-MS) can make up for the above shortcomings. PSI-MS is a rapid, cost-effective, and user-friendly method, however, the electrospray is not efficient for the ionization of PAHs [89]. In addition, high collision energy is required for PAH isomer identification. To address these issues, Zhang et al. [90] prepared COFs-paper coupled with AuNP in situ at room temperature for dual-laser-assisted PSI-MS detection of PAHs and feasible paper-surface-enhanced Raman scattering-assisted isomer identification. The LOD of the method was 0.50 ng/μL, which was nearly 300-fold higher than that of the bare paper matrix.

### 3.2. Gas Chromatography and Gas-Chromatography–Mass-Spectrometry

The basic principle of GC involves vaporizing the sample and introducing it into the column using a carrier gas. The stationary phase within the column interacts differently with each component of the sample, resulting in different efflux times and, thus, achieving separation. The detector of GC is a key component, with commonly employed detectors including a hydrogen flame ionization detector (FID), a thermal conductivity detector, and an MS detector, among others. Thanks to its high chromatographic resolution, low detection limit, and high selectivity, GC-MS has emerged as the preferred technique for the accurate quantification of PAHs in edible oils [76]. Meng et al. [36] established a method using GC coupled with electrostatic field orbit trap high-resolution mass spectrometry for the analysis of 22 PAHs (Na, Acy, Ace, F, Phe, A, Fl, P, BaA, Chr, 5-Mchr, BbFlu, BkFlu, BeP, BaP, BghiP, IP, DBahA, DBalP, DBaeP, DBaiP, and DBahP) in vegetable oils. The LODs and LOQs of the method were 0.10–0.60 μg/kg and 0.35–2.00 μg/kg, respectively, and the average recoveries were 85.1–115.4%. Internal standards are primarily used to correct the analytical errors caused by factors such as sample processing and instrument fluctuations. When the concentration range of the analyte is large or the sample matrix is complex, the use of internal standards can significantly improve the accuracy of the analysis. By providing a consistent reference, the internal standards compensate for variability in sample preparation and instrumental response, ensuring that the quantitative analysis remains robust across different experimental conditions. This is especially crucial in the analysis of polycyclic aromatic hydrocarbons in edible oils, where matrix effects and volatilization losses can otherwise lead to significant inaccuracies. To mitigate the recovery error from complex matrices and the interference from the quantitative calibration process, thereby improving accuracy, Ju et al. [56] established an isotope-dilution GC-MS method for the detection of four PAHs (BaA, Chry, BbF, and BaP) in olive oil. Using C13-labelled PAHs as an internal standard in place of deuterium PAHs resulted in improved recoveries under both low- and high-resolution MS conditions. The LODs of the method were 0.08–0.1 μg/kg, and the LOQs were 0.1–0.28 μg/kg. The GC-MS/MS method can improve sensitivity by substantially reducing the background. Zhou et al. [38] used this method to qualitatively and quantitatively analyze PAHs in edible vegetable oils. The LODs for the PAHs ranged from 0.1 to 1.0 μg/kg, with the average recoveries being between 70.0 and 110.8%, and the relative standard deviations from 2.1 to 10.2%. Compared to traditional GC, two-dimensional GC provides superior separation capacity and peak capacity. It effectively separates compounds with similar retention times in complex mixtures and provides more detailed and accurate analytical results. Drabova et al. [66] developed a two-dimensional GC-time-of-flight mass spectrometry method for the rapid determination of PAHs in vegetable oils. The recoveries ranged from 70 to 99%, the reproducibility of the method was 2–11%, and the LOQs were 0.1–0.3 μg/kg.

Benzo(b)fluoranthene, benzo(k)fluoranthene, and benzo(j)fluoranthene proved challenging to separate using conventional columns. Zhou et al. [55] chose the DB-5 MS capillary column and DB-EUPAH capillary column for the separation of PAHs in edible oils. While the DB-5 MS weakly polar capillary column performed poorly in separating benzo(b)fluoranthene, benzo(k)fluoranthene, and benzo(j)fluoranthene, it was effective for most non-polar and weakly polar organics. Conversely, the DB-EUPAH column showed superior separation of the aforementioned three PAHs.

### 3.3. Liquid Chromatography and Liquid-Chromatography–Mass-Spectrometry

Liquid chromatography (LC) is an indispensable tool for the accurate quantification of PAHs in edible oils due to its excellent separation efficiency, selectivity, sensitivity, and analysis speed. LC separates substances based on differences in the interaction of components between the mobile and stationary phases. Common detectors used in LC for the detection of PAHs in edible oils include DAD, UV, and MS detectors.

HPLC is one of the most frequently used techniques in LC. HPLC uses a liquid as the mobile phase, with single solvents of varying polarities or mixtures of solvents and buffers in different proportions pumped through a high-pressure infusion system into a column equipped with a stationary phase. For PAH determination, HPLC-FLD is still widely used, due to the natural fluorescent properties of most PAHs [3]. Zachara et al. [77] used HPLC-FLD for the determination of four PAHs (BaP, BaA, BbFA, and CHR) in vegetable oils. Payanan et al. [68] established a low-temperature clean-up-solid-phase extraction-HPLC-FLD method for determining 16 PAHs (Nap, Ace, Fl, Phe, A, F, P, BaA, Chr, BeP, BbF, BkF, BaP, DBahA, BghiP, and IP) in edible oils. However, HPLC-FLD does not allow for the determination of all 15+1 EU priority PAHs, as some of these are only weakly fluorescent or non-fluorescent, such as Acy and cyclopenta(cd)pyrene [91]. Moreover, the structural heterogeneity of such analytes prevents the simultaneous determination of all PAHs of concern in a single analysis. For the simultaneous determination of the EPA’s 16 PAHs (Nap, Ace, Fl, Phe, A, F, P, BaA, Chr, BeP, BbF, BkF, BaP, DBahA, BghiP, and IP), Zhao et al. [70] developed a SPE-HPLC-DAD-FLD method for the simultaneous determination of 16 these PAHs in vegetable oils. Acy was determined by using DAD at 228 nm, and the other PAHs were determined using FLD at optimal excitation and emission wavelengths at different times. The LODs of the method were 0.01–2.35 μg/kg. Similarly, HPLC-MS is a promising solution. However, PAHs are stable compounds and are difficult to ionize with conventional atmospheric pressure ionization techniques such as electrospray ionization and atmospheric pressure chemical ionization (APCI) [39]. Atmospheric pressure photoionization, as a novel soft ionization technique, is capable of ionizing low-polarity and non-polar analyses, thus compensating for the shortcomings of electrospray ionization and APCI in analyzing stable PAHs. Hollosi et al. [91] reported that LC-dopant-assisted atmospheric pressure photoionization tandem mass spectrometry (LC-DA-APPI-MS/MS) is capable of determining all 15+1 EU priority PAHs in the low μg/kg concentration range in a single run.

In the determination of PAHs in edible oils by LC, the separation effect of the column is an important factor affecting the peak shape and response of the target compounds. Commonly used chromatographic columns are C18 columns. A custom alkyl-bonded silica with high carbon loading is used as the column, which has excellent retention and separation of lipophilic substances [77]. In addition, the mobile phase is also an important factor in the separation process. Most researchers have used the acetonitrile/water mobile phase for the determination of PAHs by HPLC, however, injecting oil into the LC system, along with the mobile phase acetonitrile/water, will shorten the life of the column. Using acetone/water as the mobile phase keeps the pressure constant and is easier to separate from the column with better sensitivity and separation [29].

## 4. Conclusions and Perspectives

The detection and control of PAHs in edible oils are of great importance for ensuring food safety and protecting human health. This review comprehensively highlights the significant progress that has been made in the last decade in the field of sample preparation and chromatographic/mass spectrometric techniques for detecting PAHs in edible oils. Techniques such as LLE, DLLME, SPE, SPME, and QuEChERS—when combined with sophisticated detection methods like MS, GC-MS, HPLC-FLD, and HPLC-MS—have notably enhanced the accuracy and efficiency of PAH detection. However, each method presents specific advantages and limitations. For instance, while LLE is simple and reproducible, it requires significant amounts of organic solvents. In contrast, SPME offers a greener extraction approach, but at a higher cost. Among the detection techniques, GC-FID is cost-effective and environmentally friendly, but lacks the selectivity and sensitivity required for complex matrices. Conversely, GC-MS/MS provides high selectivity and sensitivity, but involves higher costs and more complex operation, demanding greater technical skill from operators. Therefore, the choice of specific sample preparation methods and detection techniques should be carefully based on practical requirements.

Despite the significant advancements, challenges still exist in terms of sensitivity, specificity, and the ability to detect low levels of PAHs in complex matrices. Future studies should focus on the development of more sensitive and selective detection methods, as well as the optimization of sample preparation techniques. This will require continued innovation and refinement in analytical techniques, along with a broader understanding of the formation and behavior of PAHs in various food matrices. The ultimate goal is to ensure the safety and quality of edible oils and, by extension, safeguard public health.

## Figures and Tables

**Table 1:** Common polycyclic aromatic hydrocarbons (PAHs) found in edible oils, presented with their acronyms and corresponding molecular structures.

**Compound**	**Acronym**	**Structure**
Benzo(a)anthracene	BaA	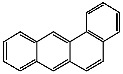
Benzo(b)fluoranthene	BbF	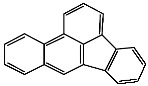
Chrysene	Chr	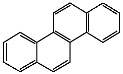
Benzo(a)pyrene	BaP	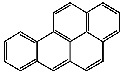
Benzo(k)fluoranthene	BkF	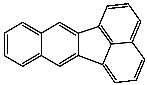
Dibenz(a,h)anthracene	DBahA	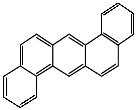
Benzo(g,h,i)perylene	BghiP	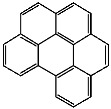
Indeno(1,2,3-cd)pyrene	IP	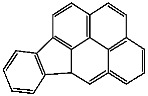
Naphthalene	Na	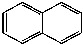
Acenaphthene	Ace	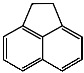
Acenaphthylene	Acy	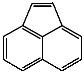
Fluorene	Fl	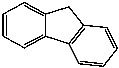
Phenanthrene	Phe	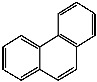
Anthracene	A	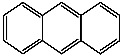
Fluoranthene	F	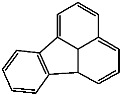
Pyrene	P	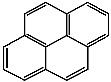
Cyclopenta(c,d)pyrene	Cpp	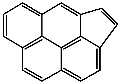
Methylchrysene	Mch	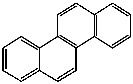
Benzo(j)fluoranthene	BjFA	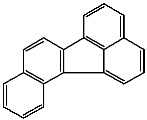
Dibenzo(a,l)pyrene	DBalP	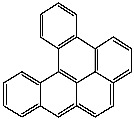
Dibenzo(a,e)pyrene	DBaeP	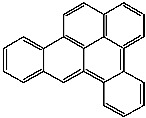
Dibenzo(a,i)pyrene	DBaiP	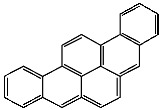
Dibenzo(a,h) pyrene	DBahP	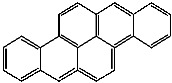
5-Methylchrysene	5-Mchr	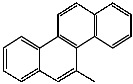
Benzo(e)pyrene	BeP	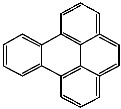
Benzo(c)fluorene	BcFl	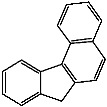

**Table 2:** Applications of LLE-based methods for extracting PAHs in edible oil samples.

Sample Amount	Analytes	Solvent Usage	Sample Preparation	Detection Techniques	LODs	Recoveries	Linearity Range	Ref.
1 g	4PAHs (BaA etc.)	Cyclohexane	LLE	HPLC-FLD	0.01–0.03 μg/kg	70.89–98.00%	0–32 μg/kg	[3]
1.5 g	BaP	ACN */ACE * (6:4, *v*/*v*)	LLE	MALDI-TOF MS *	0.1 μg/L	80.0–114.8%	0.25–50 μg/L	[34]
2.0 g	6PAHs (BaA etc.)	ACN/ACE (60:40, *v*/*v*), Fatty acid, and Na_2_CO_3_ tablet with LPIL *	EM-LPSH *	HPLC-FLD	0.02–0.19 μg/kg	80.12–103.27%	0.07–0.63–200 μg/kg	[35]
0.5 g	22PAHs (BaA etc.)	ACN	LLE	GC-MS	0.10–0.60 μg/kg	76.4–115.4%	1–100 ng/mL	[36]
2.5 mL	5PAHs (BaP etc.)	MDES *	ALLME *	HPLC-DAD	0.04–0.13 ng/g	75–88%	0.43–250 ng/g	[37]
0.5 g	23PAHs (Ba A etc.)	Hexane-saturated ACN	LLE	GC-MS/MS	0.1–1.0 μg/kg	70.0–110.8%	2–100 μg/L	[38]
0.25 g	BaP	SUPRAS *	SUSME *	HPLC-FLD	0.06 μg/kg	94–102%	0.03–5.0 ng/mL	[21]
1 g	16PAHs (Ba A etc.)	ACN	LLE	LC-APPI-MS/MS	0.006–0.156 μg/kg	77.8–106.4%	0.5–1000 ng/mL	[39]
/	5PAHs (BaA etc.)	Micellar solution	LPME	HPLC-UV	/	>95%	0.10–200 ng/mL	[40]
0.5 g	BaP	ACN	LLE	UPLC-FLD	0.2 μg/kg	81.0–97.0%	/	[29]
2.5 g	15PAHs (Ba A etc.)	ACN/ACE (60:40, *v*/*v*), C18, Florisil, and alumina-N	LPME	HPLC-FLD	0.16–0.97 μg/kg	75.0–1111.0%	0.01–90 μg/L	[41]
1 mL	14PAHs (BaA etc.)	ACN/ACE (50:50, *v*/*v*), methanolic KOH, ethanol, and tetrachloroethylene	MAE-DLLME *	GC-MS	0.2–2.7 ng/mL	84.4–101.9%	2–500 ng/mL	[42]

* ACN: acetonitrile; ACE: acetone; ALLME: air-agitation liquid–liquid microextraction; EM-LPSH: effervescent-assisted dual microextraction based on LPILs and switchable hydrophilic/hydrophobic fatty acids; LPIL: lighter-than-water phosphonium-based ionic liquids; MAE-DLLME: microwave-assisted extraction-dispersive liquid–liquid microextraction; MALDI-TOF MS: matrix-assisted laser desorption/ionization time-of-flight mass spectrometry; MDES: magnetic deep eutectic solvent; SUPRAS: supramolecular solvent; SUSME: supramolecular solvent microextraction.

**Table 3:** Applications of SPE-based methods for extracting PAHs in edible oil samples.

Sample Amount	Analytes	Sorbents	Sample Preparation	Detection Techniques	LODs	Recoveries	Linearity Range	Ref.
2 mL	5PHAs (P etc.)	CFYM *	SPE	HPLC-UV	0.37–38.5 ng/kg	60–116%	0.008–132.0 μg/L	[46]
0.5 g	16PHAs (BaA etc.)	RP-C18	LLE-SPE	GC-MS	4–110 ng/kg	87–104%	15–60,000 ng/kg	[2]
500 mg	4PHAs (BaA etc.)	C18	LLE-SPE	UHPLC-FLD	0.08–0.30 μg/kg	90.46–96.78%	0.25–20.00 ng/mL	[4]
2 g	13PHAs (BaA etc.)	C18 and Florisil	LLE-SPE	HPLC-FLD	0.10–0.38 μg/kg	81.5–107.1%	1–200 ng/kg	[8]
0.5 g	8PHAs (BaA etc.)	MIP *	LLE-SPE	UHPLC-MS/MS	/	81.40–101.14%	/	[47]
1.0 g	16PAHs (BaA etc.)	Si	UE-SPE *	GC-MS	0.06–0.13 μg/kg	84.8–115.5%	0.1–100 ng/mL	[48]
0.5 g	4PAHs (BaA etc.)	MIP	SPE	HPLC-FLD	0.10–0.19 μg/kg	89.7–109.0%	/	[49]
/	4PAHs (BaA etc.)	SiO_2_	SPE	GC-MS	/	/	/	[10]
10 g	BaP	MIL-101(Cr)	d-SPE	HPLC-FLD	0.19 ng/mL	88.8–118.8%	1–30 ng mL	[50]
1 g	BaP	SiO_2_-OCA	LLE-SPE	HPLC-FLD	0.03 μg/kg	88.0–122.3%	0.1–100 μg/kg	[33]
1 g	15PAHs (BaA etc.)	Fe_3_O_4_ @COF(TpDA)	MSPE	HPLC-DAD	0.03–0.73 μg/L	85.5–104.2%	5–100 μg/L	[51]
1 g	6PAHs (Na etc.)	mMWCNT s-ZrO_2_-C18	MSPE	HPLC-DAD	0.06–0.55 ng/g	93.5–113.2%	0.2–200 ng/	[52]
1.0 g	4PAHs (BaA etc.)	MWCNT *	d-SPE	GC-MS/MS	0.10–0.60 μg/kg	96–107%	0.1–10 μg/kg	[53]
10 mL	6PAHs (Na etc.)	SNF@Cu	SPME	GC-MS	0.1–1.2 μg/L	67–104%	0.3–500 μg/L	[54]
0.50 g	24PAHs (BaA etc.)	MIP-PAH special cartridge	LLE-SPE	GC-MS	0.1–1 μg/kg	86.0–116%	1.0–250 μg/L	[55]
4 μL	24PAHs (BaA etc.)	Florisil and C18/Z-Sep	μSPE	GC-MS	/	53–118%	5–50 ng/mL	[19]
5 g	4PAHs (BaA etc.)	EZ-POP NP dual-layer and NH_2_	LLE-SPE	GC-HRMS	0.08–0.1 μg/kg	97.5–102%	/	[56]
0.20 g	8PAHs (BaA etc.)	Silica-Amino	SPE	HPLC-FLD	0.03–0.21 μg/kg	56.5–109.4%	0.1–89.8 μg/kg	[57]
0.25 g	BaP	Fe_2_O_3_ @DA/GO	MSPE	HPLC-FLD	0.13 μg/kg	73.5–121%	/	[23]
2 mL	4PAHs (BaA etc.)	Si	LLE-SPE	GC-MS	0.27–0.85 μg/L	67.32–86.78%	/	[30]
25.0 g	13PAHs (BaA etc.)	mm-MoS_2_-GN MNPs	MSPE	GC-MS	0.10–2.50 μg/kg	70.2–112.6%	/	[58]
5 g	Naphthalene,1-methyl	/	HS-SPME	GC-MS	/	/	/	[59]
5.0 mL	4PAHs (Na etc.)	Zn5 copolymerized monolithic	μ-SPE	HPLC-UV	0.050–1.5 μg/L	86.3–101.5%	0.15–250 μg/L	[60]
500 μL	16PAHs (BaA etc.)	Florisil, a mixture of zirconia-coated silica and C18	SPE	HPLC-FLD	0.19–1.01 μg/kg	>75%	0.5–20 μg/kg	[61]
20 g	8PAHs (BaP etc.)	GOPA@Fe_2_O_3_ *	MSPE	HPLC-UV	0.06–0.15 ng/g	85.6–102.3%	0.2–200 ng/g	[62]
1 g	8PAHs (BaA etc.)	mMWCNT	MSPE	GC-MS	0.10–0.88 ng/g	87.8–122.3%	1–200 ng/g	[63]
2.0 g	8PAHs (BaA etc.)	C18 and silica	LLE-SPE	GC-MS	0.04–0.23 μg/kg	>80%	0–4 μg/kg	[5]
50 mL	9PAHs (BaA etc.)	House-made silica–alumina	LLE-SPE	HPLC-UV	0.26–1.15 μg/L	75.22–127.26%	/	[64]
2.5 g	8PAHs (BaA etc.)	Silica gel	LLE-SPE	GC-MS	1.9–3.1 μg/mL	56–84%	2–1000 μg/mL	[31]
0.5 g	BaP	[Zn(BTA)_2_] n coordination polymer	LLE-SPE	GC-MS	0.075 μg/kg	88.7–106%	0.25–10 ng/mL	[65]
1 g	16PAHs (BaA etc.)	Florisil	LLE-SPE	GC-MS	/	70.11–127.92%	5–6000 ng/mL	[28]
2.5 g	16PAHs (BaA etc.)	MIPs	SPE	GC×GC-TOFMS	/	70–99%	/	[66]
2 g	15PAHs (BaA etc.)	SDB-L	SPE	GC-MS/MS	0.01–0.06 μg/kg	55.1–105.0%	/	[67]
1 g	16PAHs (BaA etc.)	Alumina-N	LLE-SPE	HPLC-FLD	0.13–3.13 μg/kg	45.9–118.5%	0.25–150 μg/kg	[68]
500 mg	10PAHs (BaA etc.)	Carbopack Z/PDMS	DI-SPME *	GC-MS	/	/	0.10–7.93 mg/kg	[69]
2.5 g	16PAHs (BaA etc.)	ProElut C18	LLE-SPE	HPLC-DAD-FLD	0.01–2.35 μg/kg	>70%	0.05–2000 μg/kg	[70]
4 g	8PAHs (BaA etc.)	Florisil	LLE-SPE	GC-MS	0.15–0.77 μg/kg	80.6–97.8%	0.15–10 μg/kg	[71]
0.4 g	4PAHs (BaA etc.)	EZ-POP NP	SPE	GC-MS	0.1 μg/kg	86–114%	0.1–60.0 μg/kg	[72]
1 g	4PAHs (BaA etc.)	Silica	LLE-SPE	HPLC-FLD	0.06–0.12 μg/kg	>84.8%	0.2–10 μg/L	[73]
0.500 g	16PAHs (BaA etc.)	EZ-POP NP	SPE	GC-EI-MS *	0.35–1.1 μg/kg	85–98%	/	[74]
2 g	BaP	HAS	SPE	HPLC-PHRED-FLD	0.01 μg/kg	66.9–118.4%	/	[75]
5.0 g	16PAHs (BaA etc.)	3D-IL@mGO *	MSPE	GC-MS	0.05–0.30 μg/kg	80.2–115%	/	[76]
0.4 g	4PAHs (BaA etc.)	Alumina	LLE-SPE	HPLC-FLD	0.18 μg/kg	80–110%	/	[77]
20.0 g	8PAHs (BaA etc.)	Magnetic CN nanosheets	MSPE	GC-MS	0.4–0.9 ng/g	91.0–124.1%	0.5–100 ng/g	[78]

* CFYM: chicken-flaw yellow membrane; DI-SPME: direct-immersion solid-phase microextraction; GC-EI-MS: gas-chromatography–electron-impact-mass-spectrometry; GOPA@Fe_2_O_3_: phytic-acid-stabilized Fe_3_O_4_-graphene oxide; HAS: humic-acid-bonded silica; MIP: molecular imprinted polymer; MWCNT: multi-walled carbon nanotube; UE-SPE: ultrasonic extraction-SPE; 3D-IL@mGO: three-dimensional ionic-liquid-functionalized magnetic graphene oxide nanocomposite.

**Table 4:** Applications of QuEChERS methods for extracting PAHs in edible oil samples.

Sample Amount	Analytes	Sorbents	Sample Preparation	Detection Techniques	LODs	Recoveries	Linearity Range	Ref.
2 g	16PAHs (BaA etc.)	EMR-Lipids *	QuEChERS	GC-QqQ-MS *	0.06–0.13 μg/kg	66.72–112.87%	1–200 μg/kg	[85]
2 g	13PAHs (BaA etc.)	Z-Sep+-C18	QuEChERS	GC-MS	0.05 mg/kg	70.9–110%	0.01–0.5 μg/mL	[20]
2 g	16PAHs (BaA etc.)	EMR-Lipids	QuEChERS	GC-QqQ-MS	0.06–0.12 μg/kg	/	/	[86]
5 g	16PAHs (BaA etc.)	GPC	QuEChERS	GC-MS	0–37.85 ng/kg	/	/	[87]

* EMR-Lipids: enhanced matrix-removed lipids; GC-QqQ-MS: gas chromatography triple quadrupole mass spectrometry.

## Data Availability

No new data were created or analyzed in this study. Data sharing is not applicable to this article.
